# Stakeholder Perspectives on the Design of First‐In‐Human Trials for Artificial Amnion and Placenta Technology: A Qualitative Study

**DOI:** 10.1111/1471-0528.18189

**Published:** 2025-04-29

**Authors:** Angret de Boer, André Krom, Rania Kalaai, Marieke de Vries, Marije Hogeveen, Sylvia A. Obermann‐Borst, Marijn Vermeulen, M. Beatrijs van der Hout‐van der Jagt, Juliette S. van Haren, Peter Andriessen, Martine C. de Vries, Rosa Geurtzen, E. J. T. Verweij

**Affiliations:** ^1^ Department of Obstetrics Leiden University Medical Center Leiden the Netherlands; ^2^ Department of Neonatology Radboudumc Amalia Children's Hospital Nijmegen the Netherlands; ^3^ Department of Medical Ethics and Health law Leiden University Medical Center Leiden the Netherlands; ^4^ Institute for Computing and Information Sciences (iCIS) Radboud University Nijmegen the Netherlands; ^5^ Care4Neo, Neonatal Patient and Parent Advocacy Organization Rotterdam the Netherlands; ^6^ Department of Neonatal and Pediatric Intensive Care, Division of Neonatology Sophia Children's Hospital Rotterdam the Netherlands; ^7^ Department of Obstetrics & Gynecology Máxima Medisch Centrum Veldhoven the Netherlands; ^8^ Departments of Electrical Engineering and Biomedical Engineering Eindhoven University of Technology Eindhoven the Netherlands; ^9^ Department of Industrial Design Eindhoven University of Technology Eindhoven the Netherlands; ^10^ Department of Neonatology Máxima Medical Center Veldhoven the Netherlands

**Keywords:** ectogenesis, ethics, extremely premature infant, foetal viability, neonatology, parents, perinatal care, placenta, qualitative research, technology

## Abstract

**Objective:**

Artificial Amnion and Placenta Technology (AAPT), designed to improve outcomes in extreme prematurity, has shown promise in animal studies, with human trials anticipated soon. This study seeks to inform the responsible design of future trials by utilising insights from parents who experienced an (imminent) extremely premature birth and perinatal healthcare professionals (HCPs).

**Design:**

A qualitative study using individual and focus group interviews.

**Setting:**

This study was part of a Dutch study called Toward Individualised care of the Youngest.

**Sample:**

Fifteen parents who experienced an (imminent) extremely premature birth and 46 HCPs were interviewed.

**Methods:**

Eight focus‐group and five individual interviews were performed and transcribed. The transcripts were thematically analysed.

**Main Outcomes and Measures:**

The perspectives of HCPs and experienced parents on what they considered essential for human AAPT trials.

**Results:**

Analyses revealed some critical considerations represented in six themes: (1) optimise the animal model, (2) determine the goal of human trials, (3) carefully establish the research population, (4) formulate stop criteria, success criteria and outcome measures, (5) determine the role for parents during the AAPT trial, and (6) develop protocols for the trial and address logistical considerations.

**Conclusion:**

This study emphasises the critical role of stakeholder involvement in safeguarding the responsible design of human AAPT trials. Defining the trial objectives including well‐defined stop criteria and follow‐up schemes is a key element for the human AAPT trials. Establishing consensus among stakeholders is essential, as shared recommendations will facilitate alignment of expectations and promote engagement.

## Abstract 2

**Linked article**: This article is commented on by Steer pp. 1584 in this issue. To view this article visit https://doi.org/10.1111/1471‐0528.18230

## 1 Introduction

Worldwide, the main cause of perinatal mortality and morbidity is extremely premature birth, defined as birth before 28 weeks of gestation [1]. Extremely prematurely born neonates are at risk of mortality and serious physical, mental and social problems caused by the far‐too‐early transition from maternal‐placental life support to extra‐uterine life [2]. Additionally, although the offered specialised intensive care treatment after birth is crucial for survival, it can also lead to health complications, including additional iatrogenic damage, such as in bronchopulmonary disease (BPD). In light of these risks, recent research has studied techniques to postpone the transition to extra uterine physiology, such as the artificial amnion and placenta technology (AAPT) [3, 4]. Generally, AAPT technologies aim to improve clinical outcomes by limiting complications, increasing survival rates and improving quality of life for extremely premature infants [3, 4, 5]. Various models of the AAPT using lambs and piglets have been studied, showing promising results [3, 4, 6]. First in‐human trials of the technology are expected in the coming years [7].

Designing human trials for the AAPT presents complex ethical, clinical and logistical challenges. There is no consensus in the literature on how to conduct these human trials [8]. Key considerations include patient selection criteria, ethical issues in research methods such as randomisation in neonatal trials, and strategies to mitigate the risks associated with experimental technologies [8, 9]. While existing AAPT research primarily focuses on pre‐clinical technique development [5, 10], animal study challenges and outcomes [4, 5, 6, 10], and conceptual ethical and legal considerations [9, 11, 12, 13, 14], studies addressing the design of human trials for AAPT are limited [9, 11, 15]. Moreover, research incorporating the perspectives of direct stakeholders on this topic is notably absent. This study aims to fill this gap by utilising insights and experiences of parents who experienced an imminent or actual extreme premature birth and of healthcare professionals (HCPs) involved in perinatal care for extremely premature infants to guide the responsible design for the human AAPT trials.

## 2 Methods

**FIGURE 1:**
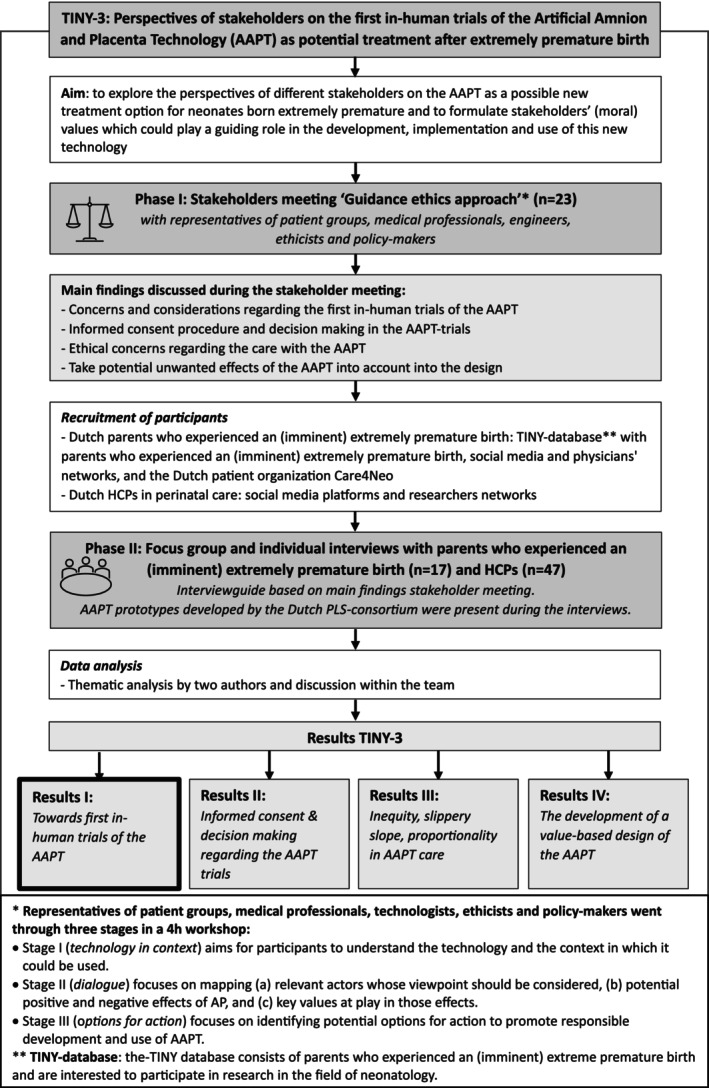
The various processes and phases of the TINY‐3 study.

This research is part of the Dutch study Toward Individualised care for the Youngest (TINY), focusing on complex decision‐making in extreme prematurity [16, 17, 18, 19]. TINY‐3 focuses on the AAPT as a potential treatment for extreme prematurity. A detailed description of the method including the COREQ‐checklist is provided in Data S1. The process of the TINY‐3 study with different phases is also displayed in Figure 1. This mixed method approach was conducted to adhere to participants' preferences and to ensure thematic saturation. The first phase consisted of a stakeholders meeting following a guidance ethics approach, summarised in Data S2 [20, 21]. To further explore and expand on the main results of the stakeholders meeting, we conducted semi‐structured focus group interviews and individual interviews (phase 2). For phase 2, inclusion criteria for participants were (1) parents who experienced an imminent or actual premature birth before 28 weeks gestation (henceforth: parents) and (2) HCPs in perinatal care. Parents were recruited through the TINY‐database. First, a general email was sent to all parents listed in the database, inviting them to participate in the study. Based on the responses, we then reached out to additional parents with diverse experiences of preterm birth and personal backgrounds to ensure a broad range of perspectives and a representative population. This article presents the TINY‐3 results on relevant considerations of parents and HCPs to safeguard the responsible design of future human AAPT trials. Both perspectives are essential because the HCPs will be the ones responsible for the care of these children, and the families would be the main characters in the trials.

One interview guide was developed for both groups based on the stakeholders meeting and the expertise of our multidisciplinary team (Data S3). Six focus group interviews with HCPs (*n* = 46) and two focus group interviews with parents (*n* = 13) were conducted, as well as five individual interviews with parents (*n* = 5). The HCPs represented various specialties, and parents had different experiences with extreme premature birth. All interviews started with an explanation of the AAPT and the Dutch context, as summarised in Box 1. The starting point was the current status of the AAPT development with the focus on the potential upcoming human AAPT trials—rather than possible future applications or clinical implementations. During the interviews, conceptual AAPT prototypes developed by the Dutch Perinatal Life Support consortium were presented to provide participants with a more tangible understanding of the technology's potential design and functionalities. Data analysis involved thematic content analysis independently performed by researchers (A.B. and R.K.) following Braun and Clarke's guidelines. This included familiarising ourselves with the data, generating initial codes, searching for themes based on these codes, reviewing the themes, defining and naming the themes and writing the manuscript [22]. An overview of the codebook with the identified themes with quotes is presented in Data S4.

BOX 1 **The Dutch context and the Artificial Placenta.**

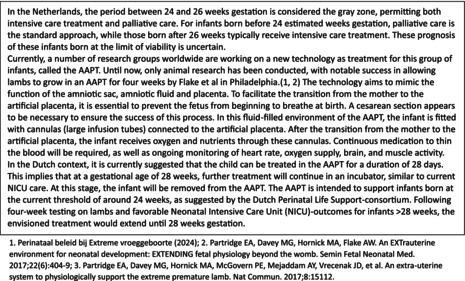

## 3 Results

Demographic information of the participants is presented in Tables 1 and 2. Results are represented in six themes derived from the data, representing the perspectives of both parents and HCPs on considerations for the upcoming human AAPT trials, namely: (1) optimise the animal model, (2) determine the goal of the first in‐human trials, (3) carefully establish the research population, (4) formulate stop criteria, cut‐off points and outcome measures, (5) determine the role for parents during the AAPT trials, and (6) develop protocols for the trial and address logistical challenges. The themes are presented in a stepwise order reflecting what should be addressed first before moving to the next phase and are supported by quotes from parents and HCPs extracted from the interviews. For each quote, it is specified whether it was stated by a HCP or a parent, along with the corresponding interview number. Additionally, supplementary quotes are provided in Table 3 to further support the findings.

**TABLE 1:** Demographic information of the healthcare professionals.

Healthcare professionals	*N* = 46
Specialty	
NICU nurse	16
Neonatologist	12
Nurse practitioner NICU	5
Maternal foetal specialist	4
Midwife/Physician assistant obstetrics	4
Obstetric nurse	3
Pathologist	1
Psychologist	1
Sex	
Female	38
Male	8
Age	
20–30	1
30–40	10
40–50	18
50–60	12
> 60	5
Working experience	
1 to 5 years	7
6 to 10 years	5
11 to 15 years	8
16 to 20 years	4
21 to 25 years	10
25+ years	12
Any experience with premature birth in personal life or social environment	
Yes	11^a^
No	35

^a^ Experiences with premature birth: < 28 weeks gestation (*n* = 3), between 28 and 37 weeks gestation (*n* = 5), or the gestation was unclear (*n* = 3). The experiences were personal, in the family or with friends.

**TABLE 2:** Demographic information of parents.

Parents	*N*
Focus group interview with	
Mother	8
Father	5
Individual interviews with	
Mother	4
Father	1
Total parents interviewed	18
Participated	
Individual	12
As couple (father and mother)	3
Age in years	
20–30	3
31–40	9
41–50	5
51–60	1
Educational level	
Secondary vocational education	7
Higher professional education	7
University education	4
**Experience with extremely premature birth**	
> 1 (imminent) extremely premature birth between GA 24 + 0 and 26 + 0	
Yes	2
No	13
Total cases	17^a^
Year of experience with extreme premature birth(s)	
2000–2008	2
2009–2016	4
2017–2024	11
GA at which extremely premature birth first threatened	
< 23 + 0	1
23 + 0 and 23 + 6	6
24 + 0 and 24 + 6	5
25 + 0 and 25 + 6	2
> 26 + 0	3
Birth between GA 24 + 0 and 26 + 0	
Yes	10
No, beyond 26 + 0	7
Multiple birth	
Yes	4
No, singleton birth	13
Initial treatment decision between GA 24 + 0 and 26 + 0^b^	
Intensive care treatment	13
Palliative comfort care	2
Parent(s) did not make a treatment decision	2
Outcome of the premature birth^c^	
Survivor(s) (incl multiples)	12
Deceased	5
Self‐reported consequences of extremely premature birth	
No consequences observed	8
Any	4

^a^ Seventeen cases of extreme premature birthincludes two couples/individual parents experiencing more than two (imminent) extremely premature births and 15 couples/individual parents experiencing one (imminent) extremely premature birth. Extremely premature birth of a multiple is recorded as one case.

^b^ This includes parents who initially opted for palliative comfort care as the treatment plan until 26 + 0 weeks gestational age, but whose infant was born after 26 weeks.

^c^ The outcome of premature births was categorised as either survivor or deceased accounting for both singleton and multiple births. For instance, the survivor(s) outcome encompasses singletons who survived or multiple births where both children survived.

**TABLE 3:** Additional quotes.

Theme	Quote
1. Optimise the animal model	‘It always remains somewhat of a gamble. You tested it on animals, but animals are not exactly like humans.’ [Parent, F7]
	‘The lambs are extracted from the AAPT after four weeks, but are full‐term by then. Infants would come out after four weeks and then what? Do they still go into the incubator?’ [HCP, F1]
2. Determine the objective of the first in‐human trials	‘There should be room to fail. […] It has to be clearly substantiated when [the trials start] with infants in critical condition to see if it works technically. It has to be accepted that things could go wrong.’ [HCP, F2]
3. Carefully establish the research population	‘Then maybe say: only if you already have a child. If the caesarean section were to cause complications, it shouldn't be the reason that you can never have another child.’ [Parent, F7]
	‘If you want to see whether this even works, you'll have to start with a group where the children are physiologically developing normally.’ [HCP, F2]
	‘Use the same criteria we currently apply to a pregnancy. At 23.5 or 24.0 weeks of gestation […] don't go below that, otherwise you won't know what you're comparing.’ [HCP, F2]
	‘Personalise per patient and parents if this would be a suitable option for the child and parents.’ [HCP, F3]
4. Formulate stop criteria, success criteria and outcome measures	‘The end point should be really clear’ [HCP, F1]
	‘…you have immediate survival, but the long term—that is of course the real obstacle, that we simply don't know. And when do you then decide: this is less favourable?’ [Parent, F7]
	‘When is it considered successful? If the infant survives? If the infants survives the neonatal phase? Or is it about the long‐term outcomes?’ [HCP, F2]
5. Determine the role for parents during the human trials with the AAPT	‘If you do not include parents, you might as well observe everything from a distance in an observatory’ [HCP, F2]
	‘Do they come for visits? Do they stay at home? […] That's another consideration, you know. Because here with us, they are allowed to visit the NICU [day and night]’ [HCP, F1]
6. Develop protocols for the trial & address logistical considerations	‘Meeting their child will be very different than with the regular treatment after birth’ [HCP, F1]
	‘Maybe the infant deteriorates significantly while still in the placenta, whereas the infant could have spent his final moments lying on his mother's chest. You need to be ahead of that moment’ [HCP, F1]
	‘This is high‐tech, you know, just like ECMO; we only have it in some centers’ [HCP, F5]

### 3.1 Optimise the Animal Model

One of the main concerns expressed by parents and HCPs was the reliability of animal research results for informing subsequent steps in human trials. They agreed that proceeding to human AAPT trials at this moment would be ‘going too fast’ and emphasised the need to conduct more animal testing: ‘*maybe you should optimize your animal model a bit more before exposing it to others*’ [HCP, F1].

The current animal models were deemed insufficient because they do not accurately reflect human pregnancy or foetal maturation. The lamb foetuses matured to full term in the AAPT, making parents and HCPs worried about how potential risks and consequences could be accurately studied. As one HCP stated: ‘w*e need to have sufficient research results to make a comparison in terms of maturity. This would allow us to say more than just: it is technically possible*’ [HCP, F2]. Furthermore, they stressed the importance of collecting data about the lambs over a longer period to collect potential long‐term effects. One parent suggested: ‘*maybe those little lambs should roam around in the meadow for a few years*’ [Parent, I3].

### 3.2 Determine the Objective of First In‐Human Trials

Particularly HCPs emphasised the importance of establishing the scientific objective before proceeding to human AAPT trials, linking it to other requirements and criteria such as establishing the research population (see Theme 3) and defining outcome measures (see Theme 4). During the interviews, potential goals emerged. Parents stated that the objective should be to test ‘*if the technology works*’ [and did not specify what parameters would include successful functioning of the technology]. HCPs framed the trials as safety trials and discussed that the aim should be ‘*data collection of benefits, harms and side effects*’ [HCP, F4]. All considered it crucial to ensure the objective of the human AAPT trials in order to gather data that is meaningful for the future population that would be treated in the AAPT.

### 3.3 Carefully Establish the Research Population

Participants stressed the need to carefully define the group of patients (mothers and infants) that would constitute the research population. Broadly, the following groups were considered eligible by HCPs: (i) based on maternal and placental factors, (ii) based on the infant's prognosis with either favourable or critical prognoses, or (iii) based on gestational age and the limit of viability.

The following groups were suggested by parents: (i) based on gestational age with ‘minimal chances of survival’ or to explore ‘interventions before 24 weeks of gestation’, (ii) based on social factors, preferring parents who already had a child to avoid ‘potential complications from a cesarean section that could affect future fertility’, or (iii) based on the level of urgency of the extremely premature birth.

No consensus was reached on which cohort would be most suitable for the AAPT trials. However, HCPs suggested personalising the decision on whether the AAPT would be a suitable option for both the parents and the infant on a case‐by‐case basis. Furthermore, the discussions tended more toward indications of the infants instead of maternal indications, supported by arguments on various levels; HCPs were mostly focused on the medical substantive arguments and parents reasoned from a broader context based on their experience with extreme premature birth.

The HCPs mentioned that the cohort that is the most ethically justifiable—for example, non‐viable infants—to involve in AAPT trials would produce the weakest research outcomes, methodologically speaking and vice versa. ‘*Infants [born after placental insufficiency] often have poorer outcomes compared to their peers. […] As a result, early experiments could yield poor outcomes, which might not be attributable to the method itself but rather to the characteristics of the infants involved*’ [HCP, F2]. Lastly, HCPs worried about the selection bias that would occur with each cohort you would choose for the human trial.

### 3.4 Formulate Stop Criteria, Success Criteria and Outcome Measures

Parents and HCPs agreed that outcome measures should be carefully formulated before trials with the AAPT start. Both groups agreed that survival should not be the only outcome measure, as it would provide a one‐sided view of the technology, and emphasised the need to examine long‐term outcomes for the infant, as well as the impact on parent–child bonding. One HCP stated: ‘*This experiment only ends, as far as I'm concerned, once the long‐term effects have been established*’ [HCP, F1]. Other outcome measures suggested by HCPs were intraventricular haemorrhage, infections, long‐term outcomes for parents in terms of mental health and bonding with their child and unspecified long‐term outcomes for the siblings.

HCPs discussed that stop criteria and success criteria should also be formulated beforehand. Criteria for stopping the trial should be established, addressing when the trial itself should be stopped and when an individual treatment within the AAPT trials should be discontinued and switched to treatment at the Neonatal Intensive Care Unit (NICU). Additionally, they argued that success criteria should be defined to determine when AAPT treatment could be deemed superior to the current NICU treatment.

### 3.5 Determine the Role for Parents During the Trial With the AAPT

Parents and HCPs stated that the autonomy of parents should be ensured during the AAPT trials by actively involving them in their infant's care. Parents argued that they should be prepared for the sight of their child in an AAPT and should be told what is expected from them, since they would not be able ‘*to hold them*’, ‘*change the diaper*’ or ‘*to care for them*’ [Parents, F7 and F8]. HCPs worried how the trials would affect parents, because parents have a significant role in current NICU‐care which also improves the bonding with their infant. One HCP stated: ‘*I think parents are a precondition [in how to design the trails with AAPT]*’ [HCP, F2].

### 3.6 Develop Protocols for the Trial and Address Logistical Considerations

Parents and HCPs expressed the need for protocols, safety procedures and addressing logistical considerations regarding AAPT trials. HCPs mostly stressed the importance of protocols for crucial moments like the transfer from the uterus to the AAPT or in case of an emergency. As one HCP stated: ‘*We need to become technically flawless in [technical procedures]. That is an absolute requirement for me*’ [HCP, F2]. Protocols and guidelines should also be developed for situations in which the transfer fails, the infant's condition in the AAPT declines or dies in the AAPT. For the first two situations, participants suggested the experiment to be stopped and treatment to be converted to standard care: ‘*It seems very important to me, as a parent, to know that […] if we see that things are deteriorating, we stop immediately and take other measures*’ [Parent, F7].

Finally, the centralisation of AAPT‐care was discussed. Particularly in the context of the trials, parents and HCPs envisioned this treatment being available exclusively at a single centre as it would allow for better control of the environment and concentration of expertise in one location. Another suggestion was to facilitate the transport of patients using the AAPT between hospitals.

### 3.7 Overall Trends

The identified themes represent overarching topics that are relevant to both HCPs and parents, showing both similarities and differences between the groups. Data collected from HCPs was observed to be noticeably richer and more detailed than that from parents. Observations indicated that participants' assumptions, interpretations or misconceptions regarding the AAPT may have shaped the findings. Although the starting point for discussion was the context of potential human trials, participants often took a broader perspective, not consistently distinguishing between the trial context and the AAPT's potential future role in care. This highlights the importance of considering care‐related concerns, which could be included as an outcome measure in future trials. Finally, a clear interconnection between the identified themes emerged, with the objectives of human trials ultimately influencing other themes.

## 4 Discussion

This study represents the first empirical study into the perspectives of key stakeholders on what they consider important to consider before human trials of the AAPT should start. The results underscore the importance of clearly defining the objective of the human AAPT trials, as it significantly influences the design and execution of each phase.

Overall, participants often assumed that the trial should aim to demonstrate the AP's superiority over the current standard of care. This is known as a superiority trial aiming to demonstrate that the AAPT provides significantly better outcomes. These outcomes should not be limited to survival but should focus more on quality of life, which is also a crucial subject for further discussion. However, parents and HCPs also discussed that the aim of the trial should be to examine the technology's safety and effectiveness. A non‐inferiority trial might be more appropriate in this context, as it aims to show that a new treatment is not significantly less effective than the standard approach (e.g., in terms of survival) while offering potential benefits, such as a lower risk of BPD or intraventricular haemorrhage.

The goals of a human AAPT trial will directly impact how the study is designed and conducted. For instance, if the aim is to compare AAPT with standard NICU care, researchers would need to select a different group of infants, use different outcome measures and follow different study protocols than if the goal were solely to assess the technology's safety [23]. In a comparison study, the most suitable participants would be infants who have a reasonable chance of survival with standard NICU care. However, including these infants poses serious ethical concerns because the risks and benefits of AAPT in humans remain uncertain.

On the other hand, safety trials might focus on non‐viable infants—babies who have no chance of survival with current medical care. While this approach could help assess the technology's safety without affecting infants who might otherwise survive, it also raises significant ethical dilemmas, such as whether it is appropriate to use AAPT in cases where survival is not possible.

The considerations outlined by our participants have notable similarities with existing legal frameworks and guidelines regulating trials, which are primarily ruled by national regulations and international standards such as Good Clinical Practice Guidelines [24, 25, 26]. These regulations describe that all preclinical research, including animal models, has to be validated to ensure safety and relevance before human trials begin [24, 25, 26]. This is reflected in the participants' concerns about the validity and translatability of the animal studies to humans, finding the evidence derived from these studies insufficient and the risk to human subjects unacceptable. So, before proceeding to human AAPT trials, consensus should be reached with key stakeholders when evidence on risks and benefits from the animal model is sufficient, which specific risks and benefits to prioritise, and when the trade‐off between these risks and benefits is considered acceptable.

In addition, the legal frameworks emphasise fairness and justice as crucial factors in selecting a research population [25]. This aligns with HCPs' concerns and considerations regarding the research group selection. Typically, three distinct research populations can be recruited for human trials: ‘healthy volunteers’, ‘seriously ill patients unable to benefit from standard of care’ and ‘patients with a stable disease’ [27, 28]. Selecting the research population depends on aspects such as the trial's scientific objective, the ‘best data’ criterion (i.e., most representative population) and the balance of risks and potential benefits [28]. Scientific justification for including certain groups is balanced against the moral obligation to minimise harm [29].

In the context of AAPT, it can be argued that there is no ‘single best population’ for human AAPT trials, which is also evident in current literature [11, 12]. For instance, the non‐viable group has been considered more ethically justifiable, though concerns remain about the potential for prolonging the suffering of entities unlikely to survive [8]. On the other hand, De Bie et al. [4] argue that a viable population may be more justifiable, particularly because the new technology is not a complete replacement for NICU care. If the AAPT technology does not work as expected, and it would be possible for the viable neonate to be transferred to an incubator, this would help mitigate some of the risks but still does not resolve the ethical complexities of selecting the most appropriate cohort for these trials.

Additionally, the situation is uniquely complex as there are two patients to consider: the mother and the infant, requiring careful consideration of both parties' health and well‐being [13]. Parents and HCPs discussed the criteria for including specific groups of infants in the human trials more extensively than the indications related to the mother or the placenta. The most proposed populations, ‘non‐viable infants’ and ‘viable with good prognosis’, are most similar respectively to the seriously ill and the stable disease population. Even if an ideal group were to be established, the results of our study suggest that HCPs and parents may hold differing views on this matter, particularly regarding the potential inclusion of the ‘viable with good prognosis’ group which should be considered carefully in terms of the willingness of parents to participate in the human AAPT trial.

Establishing the research population for the AAPT trial presents specific challenges due to the involvement of two distinct participants—the child and the mother—each governed by their own ethical and legal frameworks. Additionally, the existing shared decision‐making process with parents regarding treatment options for extremely premature infants must be preserved considering the legal requirements of the informed consent procedure [30, 31, 32]. This is further explored in another article of the TINY‐3 study [33]. Consequently, it is imperative for stakeholders to carefully assess which group constitutes the most legally and ethically responsible research population considering the infant and mother.

### 4.1 Strengths and Limitations

This is the first qualitative study on Dutch HCPs' and parents' perspectives regarding AAPT trials, offering unique insights. A varied group of participants was interviewed, enhancing the diversity of perspectives. Furthermore, the multidisciplinary nature of the research team contributes to the study's strength.

This study also has some limitations. First, the findings may be contextualised within the Dutch sociocultural milieu, its associated societal values and restrictive approach regarding the resuscitation of the most immature infant, potentially constraining the broader perspective on this technology and its human trials [34]. Nonetheless, our research underscores the significance of stakeholder engagement and demonstrates the critical importance of reflective analysis of the results. Secondly, although we purposively sampled participants from obstetrics, the majority of HCP participants had backgrounds in neonatology. Another potential limitation is selection bias, as individuals with either strong negative or positive opinions about the AAPT may have been more likely to volunteer for the study. However, thematic saturation was reached, and the results neither unequivocally endorse nor reject the AAPT, suggesting balanced results. Third, while we reached thematic saturation, it is unsure if the identified themes encompass all possible conditions. Lastly, our interpretation may have been influenced by our own perspectives. However, we took steps to mitigate this by involving a multidisciplinary team and conducting multiple rounds of discussion to ensure a more balanced and reflective analysis.

## 5 Conclusion

This study emphasises the importance of stakeholder involvement in responsibly designing human AAPT trials. Our findings underscore the importance of stakeholders reaching consensus on clearly defined objectives of human AAPT trials as these will influence critical decisions regarding the research population, study design and outcome measures. Establishing this consensus among stakeholders is essential; shared recommendations will facilitate alignment of expectations and promote engagement throughout all subsequent phases of the potential future AAPT trials.

## Author Contributions

Drs. Angret de Boer took part in designing the study, collected the data through focus groups and individual interviews, carried out the initial analyses of the data and wrote the initial draft of the manuscript. Drs. Angret de Boer had full access to all the data in the study and takes responsibility for the integrity of the data and the accuracy of the data analysis. Dr. André Krom made a significant contribution to the design of the study, took part in the collection of the data, made a substantial contribution to the analyses and interpretation of the data by participating in the discussions about the data, reviewed and revised the manuscript. Rania Kalaai was present with the collection of the data, carried out the initial analyses together with Drs. Angret de Boer and reviewed the manuscript. Prof. Dr. Martine C. de Vries, Dr. Marieke de Vries and Dr. Marije Hogeveen took part in the design of the study, collection of data, made a substantial contribution to the analyses and interpretation of the data by participating in the discussions about the data, and critically reviewed and revised the manuscript in multiple rounds of feedback. Dr. Sylvia A. Obermann‐Borst and Dr. Marijn Vermeulen made a contribution to revising our research protocol, their assistance in recruiting participants for this study, and critically reviewed and revised the manuscript. Drs. Juliette S. van Haren was present with the collection of the data and presented the prototypes of the AAPT during the interviews, critically reviewed and revised the manuscript in multiple rounds of feedback. Dr. Peter Andriessen and Dr. M. Beatrijs van der Hout‐van der Jagt critically reviewed and revised the manuscript in multiple rounds of feedback. Dr. E.J.T. Verweij and Dr. Rosa Geurtzen conceptualised and designed the study, contributed to and supervised the analyses of the collected data, and critically reviewed and revised the manuscript. Drs. Angret de Boer is the guarantor of the overall content. All authors approved the final manuscript as submitted and agree to be accountable for all aspects of the work.

## Ethics Statement

The Scientific Committee of the Leiden University Medical Centre assessed the study protocol and waived the need for ethical review (reference: 23–3052).

## Conflicts of Interest

The authors declare no conflicts of interest.

## Supporting information


Data S1.

Data S2.

Data S3.

Data S4.

## Data Availability

The data that support the findings of this study are available from the corresponding author, (E.J.T.V.), upon reasonable request.

## References

[1] World Health Organization , “Preterm Birth Factsheet 2023,” https://www.who.int/news‐room/fact‐sheets/detail/preterm‐birth.

[2] H. T. Myrhaug , K. G. Brurberg , L. Hov , and T. Markestad , “Survival and Impairment of Extremely Premature Infants: A Meta‐Analysis,” Pediatrics 143, no. 2 (2019): e20180933. 30705140 10.1542/peds.2018-0933

[3] E. A. Partridge , M. G. Davey , M. A. Hornick , et al., “An Extra‐Uterine System to Physiologically Support the Extreme Premature Lamb,” Nature Communications 8 (2017): 15112, 10.1038/ncomms15112. PMC5414058 28440792

[4] F. R. De Bie , M. G. Davey , A. C. Larson , J. Deprest , and A. W. Flake , “Artificial Placenta and Womb Technology: Past, Current, and Future Challenges Towards Clinical Translation,” Prenatal Diagnosis 41, no. 1 (2021): 145–158. 32875581 10.1002/pd.5821

[5] M. A. Coughlin , N. L. Werner , J. T. Church , et al., “An Artificial Placenta Protects Against Lung Injury and Promotes Continued Lung Development in Extremely Premature Lambs,” ASAIO Journal 65, no. 7 (2019): 690–697. 30585874 10.1097/MAT.0000000000000939 PMC11639415

[6] B. P. Fallon and G. B. Mychaliska , “Development of an Artificial Placenta for Support of Premature Infants: Narrative Review of the History, Recent Milestones, and Future Innovation,” Translational Pediatrics 10, no. 5 (2021): 1470–1485. 34189106 10.21037/tp-20-136 PMC8192990

[7] M. Kozlov , “Human Trials of Artificial Wombs Could Start Soon. Here's What You Need to Know,” Nature 621, no. 7979 (2023): 458–460.37709976

[8] E. C. Romanis , “Artificial Womb Technology and Clinical Translation: Innovative Treatment or Medical Research?,” Bioethics 34, no. 4 (2020): 392–402. 31782820 10.1111/bioe.12701 PMC7216961

[9] K. M. Werner , A. C. Baker , and M. R. Mercurio , “Unique Ethical Considerations of the Artificial Womb and Placenta: The Threshold for Patient Eligibility in Clinical Trials,” Journal of Perinatology 43, no. 11 (2023): 1335–1336. 37596392 10.1038/s41372-023-01753-x

[10] M. Yasufuku , K. Hisano , M. Sakata , and M. Okada , “Arterio‐Venous Extracorporeal Membrane Oxygenation of Fetal Goat Incubated in Artificial Amniotic Fluid (Artificial Placenta): Influence on Lung Growth and Maturation,” Journal of Pediatric Surgery 33, no. 3 (1998): 442–448. 9537554 10.1016/s0022-3468(98)90085-9

[11] S. K. Kukora , G. B. Mychaliska , and E. M. Weiss , “Ethical Challenges in First‐In‐Human Trials of the Artificial Placenta and Artificial Womb: Not all Technologies Are Created Equally, Ethically,” Journal of Perinatology 43, no. 11 (2023): 1337–1342. 37400494 10.1038/s41372-023-01713-5

[12] E. J. Verweij , L. De Proost , J. van Laar , et al., “Ethical Development of Artificial Amniotic Sac and Placenta Technology: A Roadmap,” Frontiers in Pediatrics 9 (2021): 793308, 10.3389/fped.2021.793308. 34956991 PMC8694243

[13] K. M. Werner and M. R. Mercurio , “Ethical Considerations in the Use of Artificial Womb/Placenta Technology,” Seminars in Perinatology 46, no. 3 (2022): 151521. 34893338 10.1016/j.semperi.2021.151521

[14] A. Cavolo and D. Pizzolato , “Expanding the Ethical Debate on Human Artificial Placenta Trials,” Research Ethics 21, no. 1 (2025): 9–15.

[15] M. B. van der Hout‐Jagt , E. J. T. Verweij , P. Andriessen , et al., “Interprofessional Consensus Regarding Design Requirements for Liquid‐Based Perinatal Life Support (PLS) Technology,” Frontiers in Pediatrics 9 (2021): 793531, 10.3389/fped.2021.793531. 35127593 PMC8809135

[16] A. de Boer , L. De Proost , M. de Vries , et al., “Voices of Experience: What Dutch Parents Teach Us About Values and Intuition in Periviable Decisions,” Archives of Disease in Childhood. Fetal and Neonatal Edition 110, no. 2 (2024): 171–176, 10.1136/archdischild-2024-327400. 39153843

[17] A. de Boer , L. De Proost , M. de Vries , M. Hogeveen , E. Verweij , and R. Geurtzen , “Perspectives of Extremely Prematurely Born Adults on What to Consider in Prenatal Decision‐Making: A Qualitative Focus Group Study,” Archives of Disease in Childhood. Fetal and Neonatal Edition 109, no. 2 (2023): 196–201, 10.1136/archdischild-2023-325997. 37726159

[18] L. De Proost , A. de Boer , I. K. M. Reiss , et al., “Adults Born Prematurely Prefer a Periviability Guideline That Considers Multiple Prognostic Factors Beyond Gestational Age,” Acta Paediatrica 112, no. 9 (2023): 1926–1935. 37272253 10.1111/apa.16866

[19] L. De Proost , A. de Boer , E. Verhagen , M. Hogeveen , R. Geurtzen , and E. Verweij , “Voices of Experience: Insights From Dutch Parents on Periviability Guidelines and Personalisation,” Archives of Disease in Childhood. Fetal and Neonatal Edition 110, no. 2 (2024): 165–170, 10.1136/archdischild-2024-327398. 39153841

[20] P.‐P. Verbeek and D. Tijink , Guidance Ethics Approach: An Ethical Dialogue About Technology With Perspective on Actions (ECP|Platform voor de InformatieSamenleving, 2020), 64.

[21] A. Krom , A. de Boer , R. Geurtzen , and M. C. de Vries , “Capabilities and Stakeholders—Two Ways of Enriching the Ethical Debate on Artificial Womb Technology,” American Journal of Bioethics 23, no. 5 (2023): 110–113. 10.1080/15265161.2023.2191028 37130420

[22] V. Braun and V. Clarke , “What Can ‘Thematic Analysis’ Offer Health and Wellbeing Researchers?,” International Journal of Qualitative Studies on Health and Well‐Being 9 (2014): 26152. 25326092 10.3402/qhw.v9.26152 PMC4201665

[23] F. R. De Bie , S. D. Kim , S. K. Bose , et al., “Ethics Considerations Regarding Artificial Womb Technology for the Fetonate,” American Journal of Bioethics 23, no. 5 (2023): 67–78. 10.1080/15265161.2022.2048738 35362359

[24] EUR‐Lex , “Regulation (EU) No 536/2014 of the European Parliament and of the Council of 16 April 2014 on Clinical Trials on Medicinal Products for Human Use, and Repealing Directive 2001/20/EC Text With EEA Relevance,” (2014).

[25] European Medicines Agency , “ICH E6 (R2) Good Clinical Practice—Scientific Guideline,” (2016).

[26] US Department of Health and Human Services , “Federal Policy for the Protection of Human Subjects (‘Common Rule’),” (2018).

[27] J. Shen , B. Swift , R. Mamelok , S. Pine , J. Sinclair , and M. Attar , “Design and Conduct Considerations for First‐In‐Human Trials,” Clinical and Translational Science 12, no. 1 (2019): 6–19. 30048046 10.1111/cts.12582 PMC6342261

[28] R. Dresser , “First‐In‐Human Trial Participants: Not a Vulnerable Population, but Vulnerable Nonetheless,” Journal of Law, Medicine & Ethics 37, no. 1 (2009): 38–50. 10.1111/j.1748-720X.2009.00349.x PMC2692671 19245601

[29] V. A. Miracle , “The Belmont Report: The Triple Crown of Research Ethics,” Dimensions of Critical Care Nursing 35, no. 4 (2016): 223–228, 10.1097/DCC.0000000000000186. 27258959

[30] C. Barker , S. Dunn , G. P. Moore , J. Reszel , B. Lemyre , and T. Daboval , “Shared Decision Making During Antenatal Counselling for Anticipated Extremely Preterm Birth,” Paediatrics & Child Health 24, no. 4 (2019): 240–249. 31239813 10.1093/pch/pxy158 PMC6587420

[31] J. Cummings , “Antenatal Counseling Regarding Resuscitation and Intensive Care Before 25 Weeks of Gestation,” Pediatrics 136, no. 3 (2015): 588–595, 10.1542/peds.2015-2336. 26324869

[32] S. K. Kukora and R. D. Boss , “Values‐Based Shared Decision‐Making in the Antenatal Period,” Seminars in Fetal & Neonatal Medicine 23, no. 1 (2018): 17–24. 28917833 10.1016/j.siny.2017.09.003

[33] A. de Boer , A. Krom , R. Kalaai , et al., “Healthcare Professionals' and Parental Perspectives on Human Artificial Placenta Technology‐Trials: Counselling and Informed Consent,” (2024). 10.1038/s41390-025-04051-8 40240874

[34] L. De Proost , E. J. T. Verweij , H. Ismaili M'hamdi , et al., “The Edge of Perinatal Viability: Understanding the Dutch Position,” Frontiers in Pediatrics 9 (2021): 634290, 10.3389/fped.2021.634290. 33598441 PMC7882530

