# Supplementary material for: Stakeholder Perspectives on the Design of First‐In‐Human Trials for Artificial Amnion and Placenta Technology: A Qualitative Study
Source: BJOG. 2025 Apr 29;132(11):1574–83. doi: 10.1111/1471-0528.18189 (PMC12411662; doi:10.1111/1471-0528.18189)
Supplement: Supplementary file 1 — Data S1. [file BJO-132-1574-s001.docx]

**Supplement S1: Detailed description of the methods and Coreq-checklist**

***TINY-study & study design***
This research is part of the Dutch study called Toward INdividualized care for the Youngest (TINY), initiated by three perinatal centers in the Netherlands: Erasmus MC Rotterdam, LUMC Leiden, and Radboudumc Nijmegen. While TINY-1 and TINY-2 studies focused on periviability guidelines, personalization, and parental values in decision-making surrounding extreme premature birth, TINY-3 focuses on the artificial amnion and placenta technology (AAPT) in extreme prematurity.^1-4^

As part of the TINY-3 study, a stakeholders meeting was performed following a guidance ethics approach.^5^ This approach is not primarily focused on the question whether a technology is acceptable or not, but rather starts with the question how a technology can be given a responsible place in society. In the meeting, we discussed the actors who could be affected by the implementation of AAPT (and therefore should have a say in how and under what conditions the technology should be developed and used), the potential positive and negative effects that introduction of the AAPT could have and which values would be at stake with these effects.^5^ Lastly, participants developed possible strategies to ensure the responsible development and use of the technology, specifically distinguishing options to promote key values by changes in the technology itself, options in the specific context in which the technology could be employed (e.g., organizational changes or agreements needed to promote key values) and options concerning what users of the AAPT themselves can do to promote responsible use.^5^ With the input of this stakeholder’s meeting, we performed focus group interviews and individual interviews to further explore and deepen the main findings of the stakeholders meeting:

1. Conditions and considerations regarding the human AAPT trials;
2. Considerations and concerns regarding the informed consent procedure during counselling and decision-making about the human AAPT-trials;
3. Ethical concerns regarding the care with the AAPT, such as inequality or slippery slope;
4. The AAPT design, as potential solution for some of the unwanted effects of the AAPT.

This article presents the TINY-3 results on conditions for the first in human trials of the AAPT. The Scientific Committee of the Leiden University Medical Centre assessed the study protocol and waived the need for ethical review (reference: 23-3052).

***Study participants***
Participants were recruited through the TINY-database which consists of parents who experienced an (imminent) extremely premature birth and the Dutch patient association Care4Neo, social media (e.g., LinkedIn) and the researchers’ networks. Inclusion criteria for this study were (1) working as HCP in perinatal care, or (2) being a parent who experienced an extremely premature birth before a gestational age of 28 weeks.

***TINY-database***

The TINY-database was created using Castor to include parents who experienced an imminent or actual extremely premature birth and were interested in participating in neonatology research.^1,4^ Recruitment for the database was conducted through various channels, including the Dutch patient organization Care4Neo, the Dutch platform Stille Levens - kenniscentrum Babysterfte, physician networks, social media platforms associated with participating hospitals and researchers, and by contacting parents who had consented to further communication from prior studies (PreCo study - Prenatale Counselling in extreme prematurity, CODA study)^6,7^.

To be included in the database, participants needed to meet one of the following criteria: (a) had an extremely premature birth between 23 and 26 weeks gestational age (GA) after 2010, or (b) experienced an imminent extremely premature birth between 23 and 26 weeks GA after 2010, but delivered at a later GA. Upon providing informed consent for inclusion in the database, all parents were asked to complete a brief online questionnaire. This questionnaire gathered demographic information about their experience with extremely premature birth (e.g., decision-making regarding intensive care or palliative comfort care, GA at imminent birth, GA at actual birth, and survival of the child) as well as their personal background (e.g., age, educational level, and native language). Firstly, a general email was sent to all parents listed in the database inviting them to participate in the study. Based on the responses we received, we then reached out to additional parents with different experiences of preterm birth and personal backgrounds in order to ensure a diverse population and a broad range of perspectives.

***Data collection***
Focus group and individual interviews were conducted by members of the research team with various backgrounds (PhD-student, neonatologist, maternal-fetal-medicine specialist, ethicist, psychologist). The participants all granted informed consent before starting the focus group interviews.
An interview guide was developed based on the previously performed stakeholders meeting and the expertise of our multidisciplinary team. It comprised (I) general instructions about the technique and (II) open-ended questions corresponding to the themes that were found in the stakeholders meeting. It was emphasized that the discussion should focus on the current status of the development and the most urgent topic: the potential upcoming AAPT trial, rather than on possible applications of implementation in clinical care. During the interviews, prototypes developed by the PLS consortium were present to provide participants a clearer, more concrete understanding of what the technology could look like.

***Data analysis***
Results were analyzed and coded independently by two authors (AB, RK) in Atlas.ti, using thematic content analysis following Braun and Clarke’s guidelines.^8^ This process included becoming familiar with the data, generating initial codes to develop a codebook, identifying patterns and themes within the data, reviewing and updating codes and codebook, and defining and naming the themes. The codebook and analysis were discussed within the team and adjusted in multiple rounds until agreement was reached.

***Manuscripts TINY-3***

The data collected from the interviews was extensive and multi-faceted, addressing various ethical considerations regarding the AAPT and the upcoming human trials. To thoroughly analyze and present these findings, we divided the data into four distinct manuscripts, each corresponding to key themes outlined in the interview guide and additional themes that emerged during our analysis.

Given the volume and complexity of the data, as well as the ethical implications raised throughout the discussions, four critical topic are separated into 4 manuscripts (see also figure 1) allowing for thorough examination of each critical theme. This approach ensures that fundamental ethical questions and concerns identified during the interviews receive the necessary attention and discussion.

The first paper focuses on the conditions surrounding the initiation of first-in-human trials.

**COREQ (Consolidated criteria for Reporting Qualitative research)- checklist^9^

Name**: Angret de Boer
**Affiliation**: Department of Obstetrics, Leiden University Medical Center, The Netherlands; Radboud University Medical Center Nijmegen, Radboud Institute for Health Sciences, Amalia Children’s Hospital department of neonatology, The Netherlands
**Research:** Considerations from parents and professionals to inform responsible design of first-in-human artificial amnion and placenta trials

| Item | Topic | Description |
| --- | --- | --- |
| **Domain 1: Research team and reflexivity** | | |
| 1. | Interviewer/ facilitator | A.H.A. de Boer |
| 2. | Credentials | MD, PhD-student |
| 3. | Occupation | Junior doctor, PhD-student |
| 4. | Gender | Female |
| 5. | Experience and Training | She received formal education and training in conducting interviews. |
| *Relationship with participants* | | |
| 6. | Relationship established | No |
| 7. | Participant knowledge of interviewer | Educational background, occupation and reasons for doing research |
| 8. | Interviewer characteristics | Reasons and interests in the research topic |
| **Domain 2: Study design** | | |
| *Theoretical framework* | | |
| 9. | Methodological orientation and theory | Content thematic analysis |
| *Participant selection* | | |
| 10. | Sampling | Purposive sampling |
| 11. | Method of approach | Social media of the patient organization Care4Neo and Stille levens, the researchers, and the hospitals (LUMC, Erasmus MC, Radboudumc) |
| 12. | Sample size | 46 |
| 13. | Non-participation | Research supervisors (EV, RG, MCdV, AK, MdV, MH) |
| *Setting* | | |
| 14. | Setting of data collection | Focus group interviews and individual interviews |
| 15. | Presence of non-participants | Yes, observer for time management and researcher from TU Eindhoven for the prototypes |
| 16. | Description of sample | Healthcare professionals working in perinatal care and parents who experienced a (imminent) extremely premature birth between 24-28 weeks of gestation. |
| *Data collection* | | |
| 17. | Interview guide | Semi structured, with open-ended questions |
| 18. | Repeat interviews | No |
| 19. | Audion/ visual recording | Audio-recorded |
| 20. | Field notes | No |
| 21. | Duration | 1,5-2 hours |
| 22. | Data saturation | Yes |
| 23. | Transcripts returned | No |
| **Domain 3: Data analysis and findings** | | |
| *Data analysis* | | |
| 24. | Number of data coders | 2 independent coders, discussion within the research team |
| 25. | Description of the coding tree | As recorded in Table 2. |
| 26. | Derivation of themes | Themes were derived from the data |
| 27. | Software | Atlas.ti |
| 28. | Participant checking | Yes the last two versions of the manuscript |
| *Reporting* | | |
| 29. | Quotations presented | Yes |
| 30. | Data and findings consistent | Yes |
| 31. | Clarity of major themes | Yes |
| 32. | Clarity of minor themes | Yes |

**References**

1. de Boer A, De Proost L, de Vries M, Hogeveen M, de Vries MC, Verweij E, Geurtzen R. Voices of experience: what Dutch parents teach us about values and intuition in periviable decisions. Arch Dis Child Fetal Neonatal Ed. 2024.

2. de Boer A, De Proost L, de Vries M, Hogeveen M, Verweij E, Geurtzen R. Perspectives of extremely prematurely born adults on what to consider in prenatal decision-making: a qualitative focus group study. Arch Dis Child Fetal Neonatal Ed. 2023.

3. De Proost L, de Boer A, Reiss IKM, Steegers EAP, Verhagen AAE, Hogeveen M, et al. Adults born prematurely prefer a periviability guideline that considers multiple prognostic factors beyond gestational age. Acta Paediatr. 2023;112(9):1926-35.

4. De Proost L, de Boer A, Verhagen E, Hogeveen M, Geurtzen R, Verweij E. Voices of experience: insights from Dutch parents on periviability guidelines and personalisation. Arch Dis Child Fetal Neonatal Ed. 2024.

5. Verbeek P-P TD. Guidance Ethics Approach: An ethical dialogue about technology with perspective on actions. . The Hague: ECP | Platform voor de InformatieSamenleving,; 2020. 64 p.

6. Geurtzen R, Draaisma J, Hermens R, Scheepers H, Woiski M, van Heijst A, Hogeveen M. Various experiences and preferences of Dutch parents in prenatal counseling in extreme prematurity. Patient Educ Couns. 2018;101(12):2179-85.

7. Geurtzen R, van Heijst AFJ, Draaisma JMT, Kuijpers L, Woiski M, Scheepers HCJ, et al. Development of Nationwide Recommendations to Support Prenatal Counseling in Extreme Prematurity. Pediatrics. 2019;143(6).

8. Braun V, Clarke V. What can "thematic analysis" offer health and wellbeing researchers? Int J Qual Stud Health Well-being. 2014;9:26152.

9. Tong A, Sainsbury P, Craig J. Consolidated criteria for reporting qualitative research (COREQ): a 32-item checklist for interviews and focus groups. Int J Qual Health Care. 2007;19(6):349-57.
