# Supplementary material for: Stakeholder Perspectives on the Design of First‐In‐Human Trials for Artificial Amnion and Placenta Technology: A Qualitative Study
Source: BJOG. 2025 Apr 29;132(11):1574–83. doi: 10.1111/1471-0528.18189 (PMC12411662; doi:10.1111/1471-0528.18189)
Supplement: Supplementary file 2 — Data S2. [file BJO-132-1574-s004.docx]

**Supplement S2: Summary of the report of the 'Workshop on Guidance Ethics'**
*Disclaimer: This is a summary of the full report, translated from Dutch.*

On April 7, 2023, a workshop on guidance ethics was conducted concerning the development of an artificial amnion and placenta technology (AAPT) as a potential future treatment for extreme prematurity at the limit of viability. The objective of the workshop was to facilitate a structured dialogue with relevant stakeholders regarding the ethical aspects surrounding the development of this technology.
Using the Guidance Ethics Approach, the workshop explored the potential effects of developing and using the AAPT, as well as the values involved. Subsequently, the participants identified possible action options to enhance positive effects and mitigate any negative impacts on significant ethical values.
A total of 23 individuals participated in the workshop, including a moderator and subgroup moderators. Participants included individuals with various medical and technical backgrounds, ethicists, legal experts, parents, representatives from the patient organization Care4Neo, and policymakers. The workshop was facilitated by a moderator from the Department of Ethics and Law in Healthcare at LUMC.

**Guidance Ethics Approach and Workshop Structure**
The Guidance Ethics Approach was developed by the Ethics and Digitalization Working Group of ECP (Platform for the Information Society). The group was chaired by Peter-Paul Verbeek (University of Twente) and Daniël Tijink (ECP). In 2019, they published the "Guidance Ethics Approach."^1^ These workshops typically last 3.5 to 4 hours and follow a standardized structure that allows for the exploration of various aspects of the case.

A guidance ethics workshop consists of the following phases:

**Phase 0: Introduction**

- Discussion of the objectives of the Guidance Ethics Approach and an explanation of the underlying concepts.

**Phase 1: Technology in Context**

- An explanation of the technology and the context in which it may be used. Participants have the opportunity to ask clarifying questions.

**Phase 2: Dialogue**

- A brief round in which workshop participants identify actors who would be involved with the technology.
- Participants discuss potential positive and negative effects that the technology may have.
- Participants identify values that play a role in those effects, considering which values are likely to be promoted and which may be at risk if the technology is used.

**Phase 3: Action Options**

- Participants explore options for action to enhance the positive effects of the technology and to prevent or minimize negative effects. This helps protect or promote important values.
- Options for action are explored from three perspectives:
  - What can be changed about the technology itself?
  - What can be changed in the physical, social, or institutional environment in which the technology may be used?
  - What responsibilities do users of the technology have regarding its responsible use?

Some minor adjustments were made to certain components compared to the standard application of the approach.

**Phase 1: Technology and Context**
**Case: Technology in Context**
To understand the ethical aspects involved in using a technology and how to handle it responsibly, it is essential to know more about the technology itself and the context in which it will be (potentially) used.

In preparation for the workshop on April 7, 2023, participants received a case description regarding the AAPT. At the beginning of the workshop, a brief presentation about the AAPT was given about the context in which this technology could initially be used; after extremely premature birth at 24 weeks with a duration of 4 weeks (till 28 weeks). Following the presentation, participants could ask clarifying questions.

**Phase 2: Dialogue**
During the dialogue phase, participants discussed who would be involved or could be involved in the development and potential use of the AAPT. They also deliberated on the positive and negative effects of using the AAPT, along with the important values associated with those effects. These values were explicitly identified to ensure they are adequately considered in the potential use of the AAPT.

**Actors**
In addressing the topic of "actors," the question was raised about who is involved or could be affected by the use of the AAPT. The workshop participants already represented various actors. They were asked to identify other stakeholders who should also be involved. Examples of actors mentioned from three out of ten categories include:

| **Category** | **Identified Actors** |
| --- | --- |
| Family | Pregnant individuals, healthy pregnant individuals, partners, the child/foetus, siblings, extended family (e.g., grandparents), healthy full-term children, former patients (particularly those with complications), parents of twins, childless couples, patient organizations representing parents and patients. |
| Care | Various physicians: obstetricians, neonatologists, paediatricians from follow-up clinics, ophthalmologists (regarding the risk of retinopathy), general practitioners, perfusionists, nurses, psychologists, midwives, medical social workers, spiritual caregivers, pedagogues, professional associations such as the Dutch Association for Obstetrics and Gynaecology (NVOG) and the Dutch Society for Paediatrics (NVK). |
| Science | Researchers, sociologists, ethicists, physiologists, trial participants, laboratory animals, Medical Ethics Review Committees (METC). |

Table 1: Actors as identified by participants in the workshop on April 7, 2023.

**Effects**
The standard inquiry in this section pertains to the potential effects of using the technology, both positive and negative. After merging and removing some identified effects, a total of 48 possible effects were retained, consisting of 12 positive and 36 negative effects. For example, the reported positive and negative effects related to the child are as follows:

| **Positive** | **Negative** |
| --- | --- |
| CHILD | |
| 1. Fetal physiology is maintained |  |
|  | 1. Medicalization of birth and the postpartum period. |
| 1. Reduced damage to organ systems. | 1. Increased damage to organ systems. |
|  | 1. Uncertainty/unknown outcomes, leading to the need to make decisions without being able to base them on known outcomes. |
| 1. Preventing (over)extension, which often occurs when a child has spent a prolonged period in an incubator. | 1. Poorer motor development of the child and the absence of the effects of maternal movement. |
| 1. Fewer painful procedures compared to the incubator, such as frequent needling, leading to reduced stress for the newborn. |  |
| 1. Improved quality of life | 1. Reduced quality of life |
| 1. Improved survival chances (life-saving) | 1. A surviving baby is not always "healthy" or without significant complications. |

**Table 2:** Positive and negative effects of the use of the AAPT on the child, as identified by participants in the workshop on April 7, 2023.

**Values**
The next step in the Guidance Ethics Approach is to identify the values associated with the previously mentioned effects. Participants identified a total of 35 values. Without exception, multiple values were linked to each effect. The values specifically associated with the aforementioned effect 5 are listed in the table below:

| **Negative effect** | **Values (Alphabetical)** |
| --- | --- |
| 5. Uncertainty/unknown outcomes, leading to decisions made without being able to base them on known outcomes. | Autonomy, freedom, health, solidarity, welfare of the child and parent. |

**Table 3:** Values that participants indicated play a role in a selection of effects related to the development and use of the AAPT.

In **Table 4**, all discussed values are listed separately (alphabetically).

| **Waarden** | | |
| --- | --- | --- |
| (Right to) an open future  Ability to care  Accessibility  Achieving maximum potential  Affection  Autonomy / Self-determination  Bodily integrity  Bonding/attachment  Carefulness  Connectedness  Coping  Cost-efficiency  Development | Enjoyment  Equality of opportunity  Fairness  Freedom  Frugality  Functioning  Good parenting  Health  Healthy development  Meaningfulness  Participation  Patience  Proportionality | Protection (of your child)  Quality of life (parents, child)  Relationships/contact  Reproductive autonomy  Resilience  Safety  Sense of responsibility  Solidarity  Welfare (child, parent, pregnant individual) |

**Table 4:** Values discussed during the workshop on April 7, listed alphabetically.

**Phase 3: Action Options**

In this phase, the focus is on identifying concrete actions that can be taken to enhance positive effects and minimize negative effects. By considering action options, important values can be better protected.

A brief explanation was provided regarding the various 'levels' at which responsible action options can be contemplated concerning the use of the AAPT as a treatment for extreme prematurity. This may involve the design of the technology, adjustments to the (physical, social, or institutional) environment in which the AAPT would be used, and the behavior of users. Participants then worked in subgroups to brainstorm action options for the positive and negative effects listed.

In the table below, the options for action that were identified are listed:

| **Handelingsopties** | | | |
| --- | --- | --- | --- |
| **Effect** | **Technology** | **Environment** | **User** |
| 5. Uncertainty/unknown outcomes, leading to decisions made without being able to base them on known outcomes. | - Continue developing the technology based on interim results (including evaluation moments). | - Establish boundaries in research development regarding inclusion and exclusion criteria for both child and mother, formulate clear stop criteria. - Ensure long-term follow-up of included participants, evaluate these results before progressing to the next phase of research, ensure broad follow-up across various domains. - Promote transparency and the use of 'honest data,' ensuring the technology is not portrayed in a more favorable light than it is. - Make decisions clear and comprehensible, share them in a timely manner, and ensure transparency. |  |

**Table 5: Action options for effect 5 mentioned during the workshop**

**Reference:** 1. Verbeek P-P TD. Guidance Ethics Approach: An ethical dialogue about technology with perspective on actions. . The Hague: ECP | Platform voor de InformatieSamenleving,; 2020. 64 p.
