# Supplementary material for: Stakeholder Perspectives on the Design of First‐In‐Human Trials for Artificial Amnion and Placenta Technology: A Qualitative Study
Source: BJOG. 2025 Apr 29;132(11):1574–83. doi: 10.1111/1471-0528.18189 (PMC12411662; doi:10.1111/1471-0528.18189)
Supplement: Supplementary file 3 — Data S3. [file BJO-132-1574-s003.docx]

**
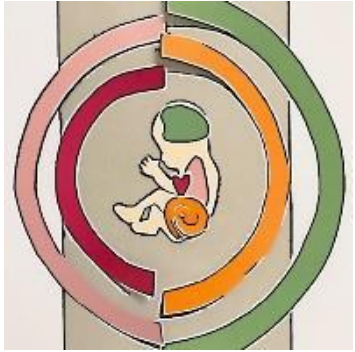
 Supplement S3: Interview Guide TINY-3 (*translated from Dutch to English)***

**Introduction:**

- Explanation about how the focus group interviews will be performed, some regulations and practical matters
- Informed consent and demographic information
- Introduction to the topic: treatment of extreme prematurity and the current status of the development of the artificial amnion and placenta technology, which is the study phase before the first-in-human trials might start (i.e., before the first baby is placed in the artificial amnion and placenta technology). It can/may still be decided that this is not desirable.

1. **Ethical Considerations for the Application of the Artificial amnion and placenta technology:**
   - Is the development of the artificial amnion and placenta technology a desirable advancement? Why or why not?
   - What are your primary ethical dilemmas associated with the application of the artificial amnion and placenta technology in humans? Why?
   - What ethical conditions must be met before first in human trials with the artificial amnion and placenta technology could be started?
     - What actions are necessary to fulfill these conditions?
2. **Counseling and Decision-Making at the Individual Level**
   - Interview with parents:
     - What personal arguments, reasons, or circumstances might influence your decision to participate or not participate in such a trial?
     - What support or information would you need to make an informed decision about participating in the study involving the application of the artificial amnion and placenta technology in humans?
   - Interview with Healthcare Professionals:
     - What minimum standards do you believe counseling should meet to enable parents to make a decision about participating in the first study involving the application of the artificial amnion and placenta technology in humans?
3. **Design of the Artificial amnion and placenta technology:**
   - Which aspects of current daily care for infants between 24-28 weeks of gestation in the NICU do you consider essential to retain in an artificial amnion and placenta technology?
   - What negative aspects of the current daily care for infants between 24-28 weeks in the NICU does the artificial amnion and placenta technology have the potential to improve or resolve?
   - What minimum conditions must the design meet before we can further develop or utilize the artificial amnion and placenta technology?

*Present physical and digital prototypes.*

- - Do you still agree with the conditions you previously mentioned, or do you have any additions?
