# Supplementary material for: Stakeholder Perspectives on the Design of First‐In‐Human Trials for Artificial Amnion and Placenta Technology: A Qualitative Study
Source: BJOG. 2025 Apr 29;132(11):1574–83. doi: 10.1111/1471-0528.18189 (PMC12411662; doi:10.1111/1471-0528.18189)
Supplement: Supplementary file 4 — Data S4. [file BJO-132-1574-s002.docx]

| Themes (conditions) extracted from the data | *Subthemes extracted from the data* | Parental perspectives and their suggestions with corresponding quotes | healthcare professionals’ PERSPECTIVES and their suggestions with corresponding quotes |
| --- | --- | --- | --- |
| 1. Optimize the animal model | *The current animal model is not sufficient, more tests are necessary* | The development is going too fast. More tests with (other) animals are necessary  “*It would have been nice if a few more lambs had been born*” | The animal model should be optimized first, before exposing humans to this technology  *"It always remains somewhat of a gamble. You tested it on animals, but animals are not exactly like humans."* |
|  |  |  | More knowledge is necessary beyond technical feasibility  *"…we need to have sufficient research results [to compare animals and humans] in terms of maturity. We need to be able to say more than: it is technically possible”* |
|  | *The current animal model is not comparable to humans* | Animals are not the same as people  "*...and [it may be good to test the AAPT] on an animal that has more similar characteristics [as a human]"* | This animal model cannot be compared to the human situation, the maturation of the lambs is not comparable to that of a human  *"The lambs are extracted from the AAPT after four weeks, but are full-term by then. Infants would come out after four weeks and then what? Do they still go into the incubator?"* |
|  | *The current animal model should collect more long term data before moving forward to human trials* | Let the lambs live to get more information about the consequences  *"..let those lambs roam in the meadow for a few more years"* | To collect data over longer time to identify the consequences of the treatment  *"You have no idea (…) whether the risks at 28 weeks are actually lower than before. They may have already sustained damage in the artificial womb, and this could only be worsened by birth at 28 weeks."* |
| 2. Determine the objective of the first in-human trials |  | To test the technique.  *"All the what if’s"* | Should be a safety trial  *“There should be room to fail. […] It has to be clearly substantiated when [the trials start] with infants in critical condition to see if it works technically. It has to be accepted that things could go wrong.”* |
|  |  |  | To collect more data about benefits, harms and side effects  *"Otherwise, we might end up with children who survive the initial period, but die from cardiovascular diseases by the time they are fifteen"* |
| 3. Carefully establish the research population | *Start trials with a group of mothers and infants that are born based on problems with the* ***pregnancy, placenta or pregnant person.*** |  | Suggestions for maternal indication:  *“A child in a situation where the mother is at risk, but the child is actually doing well”*   - Placenta insufficiency   - Contra: infants with bad prognosis - Extreme premature birth with a poor prognosis after intraventricular hemorrhage - If an abdominal cerclage is not an option/failing - The mother’s illness, e.g., pre-eclampsia   *Arguments:*   - The cesarean section would be justifiable - More time to counsel parents about the trial |
|  | *Start human trials with a group of infants that have an* ***estimated*** ***good prognosis*** |  | Suggestions for a group of infants with a **good prognosis**  “*If you want to see whether this even works, you'll have to start with a group where the children are physiologically developing normally*”   - Having the best birthweight, are most matured, and are born with a spontaneous premature birth - Are not small for gestational age, have no congenital abnormalities, no maternal sepsis, intra-uterine infections or pre-eclampsia - With the least risk on intraventricular hemorrhage   *Arguments:*   - To give the technology a chance to prove itself - Most comparable to the population currently offered early intensive care treatment   *Contra arguments:*   - The birth of this group of infants is often very quick, so there would probably be little time to counsel these parents - There is less to gain but more to lose with a good prognosis |
|  | *Start human trials with a group of infants that have an* ***estimated poor prognosis*** |  | Suggestions for a group of infants with a **poor prognosis**:  *“I would start with the group with the poor prognosis”*   - The alternative is death of the infant - Placental insufficiency leading to severe fetal growth restriction - The group of infants that, in the current situation, would only be treated beyond 26 weeks gestation/the grey zone   *Arguments:*   - To give an infant a last chance - No ‘better’ alternative for the infant |
|  | *Start human trials with a group of infants that are born* ***at 24 weeks gestation or later*** | Suggestion to start human trials with infants that are born at 24 weeks gestation.   - They have little chance of survival   *“I would only choose it if you're on the limit of viability”* | Suggestion to start human trials with infants that are born at 24 weeks gestation or later:  *“Use the same criteria we currently apply to a pregnancy. At 23.5 or 24.0 weeks of gestation […] don't go below that, otherwise you won’t know what you're comparing”*   - The ‘best’ infants, with the best prognosis, with the least risk for an intraventricular hemorrhage - The prognosis is not disastrous - The infant has the same conditions as in the situation without this experimental treatment - Specifically when the chance on improved outcomes is better with the AAPT than with the current Neonatal Intensive Care Unit (NICU)-treatment   *Arguments:*   - The technology has the potential to surpass current NICU treatments |
|  | *Start human trials with a group of infants that are born* ***before 24 weeks*** *gestation* | Suggestion to start human trials with infants that are born before 24 weeks gestation:   - Otherwise they have no chance of survival   *“I think the discussion should focus on: are you going to treat even more vulnerable children? […] or will you explore what could be possible under 24 weeks? [The limit of viability] will likely become more flexible over time”* | Suggestion to start human trials with infants that are born before 24 weeks gestation:  *“So, you would opt for AAPT in case the [NICU treatment} would almost certainly be a poor prognosis. In the Netherlands, this would mean children who are just before the limit of viability, perhaps a few days prior, who would not survive without this treatment”*  Meaning:   - Infants who currently are not treated and would otherwise pass away   *Arguments:*   - The chances for the group of infants born between 24-26 weeks are improving - These infants will currently not be offered early intensive care treatment in our country and will therefore not survive.   *Contra arguments:*   - Potentially prolonging of suffering and/or delaying the inevitable - It is not comparable to the group of extremely premature infants who currently can be treated and will be the group of infants that would receive the treatment with the AAPT if it would be implemented - It’s a challenging situation because, in the Netherlands, we adhere to a 24-week viability limit, whereas in some other countries, the threshold is set at 22 weeks. As a result, infants born before 24 weeks are already being treated in certain parts of the world, meaning the argument that these children wouldn’t survive does not hold true everywhere. |
|  | *Start human trials with a group that is* ***not in an emergency situation*** *of extremely premature birth* | Suggestion to start human trials with a group of parents and infants that are not in an emergency situation of extremely premature birth, so parents can be approached in time.  *“When do you inform parents? Because I don’t think it would have been possible in my case. I arrived at the hospital and had to hold back the contractions. She was almost born by the time I got there”* | Suggestion to start human trials with a group of parents and infants that are not in an emergency situation of extremely premature birth, parents can be approached in time  *“If you're going to perform a cesarean section, you typically do not have much dilation. So […] if you have a bulging bladder and a infant that is almost being born, I do not think you will be able to proceed with the cesarean section”* |
|  | *Start human trials with a group of* ***parents that already have a child*** | Suggestion to start human trials with a group of parents who already had a child before  *“Only if you already have a child. If a C-section were to cause complications, it should not result in the inability to have another child in the future”* *Arguments*   - Balancing of the potential consequences of the necessary caesarean section on potential future pregnancies or children may differ |  |
|  | ***Personalize*** *if the AAPT trial is a suitable option* |  | Suggestion to personalize on a case by case basis if the human trials with the AAPT is a suitable option:  *“Personalize per patient and parents if this would be a suitable option for the child and parents”* |
|  | *Minimize the selection bias* |  | Concerns:   - Good prognosis: *“* *You will already be doing a pre-selection […]. I assume that those with significant morbidity would not be selected for this [treatment]. So, you’re going to be selecting the best candidates. [...] And then, if it works for those best candidates? Well, then you're already facing challenges”* - Poor prognosis: *“the mortality and morbidity caused by the selection of the population may be caused by your participants selection, instead of the technology”* |
| 4. Formulate stop criteria, success criteria and outcome measures | *Formulate stop criteria* |  | Before starting the human trials, it should be determined when to stop the human AAPT trials, potentially meaning the end of the development of the AAPT *“The end point should be really clear”* |
|  |  |  | Before starting the human trials, it should be determined when to stop AAPT treatment and transition the patient to incubator-based care *“You really can't put parents through that—continuing [the treatment] for four weeks and then coming to the conclusion that it's time to stop [with the treatment] after all”* |
|  | *Formulate success criteria* |  | Before starting the human trials, clear criteria should be established to determine when the experimental treatment has succeeded and can progress to the next phase of testing.   - Successful treatment in the AAPT until 28 weeks - Long-term consequences should be looked at - Survival   *“When is it considered successful? If the infant survives? If the infants survives the neonatal phase? Or is it about the long-term outcomes?”* |
|  | *Formulate outcome measures* | It's important to consider the long-term consequences, not just mortality  *“Survival is one aspect, but the long-term outcome... that's the real challenge—we simply don't know. And when do you decide to say: it's not going as well as hoped?”* | It's important to consider the long-term consequences, not just mortality or short term morbidity  *“..that's the risk—that survival becomes the only endpoint”* |
|  |  |  | *“Compare all aspect with the [current] neonatal intensive care treatment”* |
|  |  | Important to look at the long term outcomes of parents:   - “*The psychological impact on parents*” - “*Bonding with your infant*” | Important to look at the long term outcomes of parents:   - *“The impact on the maternal mental health, not being able to do anything with your infant*” - Bonding with the infant |
|  |  | Important to look at the long term outcomes of the infant:   - Long-term consequences   *“Has it already been decided how long the follow-up for the first group will be? The question they couldn’t answer for me during the threatening preterm birth was: how will my child be doing when they're eighteen?”* | Important to look at the long term outcomes of the infant:   - Long-term consequences - Bonding with parents   *“Even subtle changes, like epigenetic shifts or the risk of disease later in life, we just don't know. It's so subtle that I don't think you can ever uncover that in those initial organ studies [in the animal models]”* |
|  |  |  | Important to look at the consequences for the siblings  *“And what about the [impact on] brothers and sisters? How do you explain that to them? [...] "Our little brother was in an egg"”* |
| 5. determine the role for parents during the human trials with the AAPT | *Ensure the involvement of parents in the AAPT treatment during the AAPT trial* | Ensure the autonomy and self-determination of parents in treatments involving the AAPT by actively involving them in the care of their infant.  *“So, allowing them to do as much as possible themselves [in the care for their child] and have as much autonomy as they can”* | Involving parents in the AAPT treatment during the trial is a condition before starting the human trials.  *“If you do not include parents, you might as well observe everything from a distance in an observatory”* |
|  |  | Consider how parents can contribute to the care of their infant during treatment with the AAPT.  *“In the incubator, you can care for [your infant]. But when they are in an AAPT, the role of the parents changes significantly. That’s something to consider... It was really wonderful to change diapers”* | Consider how parents can contribute to the care of their infant during treatment with the AAPT, ensure moments of contact are possible, ensure parental presence during AAPT.  *“Do they come for visits? Do they stay at home? […] That’s another consideration, you know. Because here with us, they are allowed to visit the NICU [day and night]”* |
|  |  | Appeal to the feelings of motherhood and fatherhood by fostering emotional connections and encouraging bonding, even when parents are unable to physically touch or care for their child.  *“I would also want to have a therapist alongside the parents because the infant is already born, but they have less contact. […]. It is important to support the connection and attachment, addressing the maternal and paternal feelings as well.”* |  |
|  | *Consider the changing role for parents* | The role of parents will be different during a human trials with the AAPT:   - Parents cannot take care of their infant like in current NICU care. Taking care of their infant is an important experience for parents. - Parents cannot provide kangaroo care to their infant. | The role for parents will be different, this may also change grief for their infant.  *“Contact with the infant, if you can see them [in the AAPT], is completely different than when they are still in the womb [...] And then, after three weeks of observing your infant and almost holding them in your hands, to lose them afterward would make the pain even more profound.”* |
| 6. Develop protocols for the trial & address logistical considerations | *Develop a protocol for the transfer from the uterus to the AAPT as part of the human AAPT trials* |  | The transfer of the infant from the pregnant person to the AAPT should be meticulously planned, with each step carefully considered to ensure the safety and well-being of both the infant and the parent   - The medical interventions needed should be executed flawlessly - Define what to do if something goes wrong - Define how to involve parents in this process   “*Meeting their child will be very different than with the regular treatment after birth*” |
|  | *Develop a protocol for failing of the transfer from the uterus to the AAPT as part of the human AAPT trials* |  | The child should be treated with routine regular care if the transfer fails. Clear criteria to define an unsuccessful transfer need to be determined in advance.  “*But if the transfer to the AAPT doesn't succeed, the infant should diverted to the standard treatment arm*” |
|  | *Develop a protocol for failing of AAPT treatment during the trial* | If AAPT treatment fails, AAPT treatment should be stopped and be diverted to routine NICU care.  “*If it is necessary to take the infant out the AAPT, that should always be prioritized. The infant should then be placed in an incubator for further support*” | If AAPT treatment fails, the AAPT treatment should be stopped and treatment should be diverted to routine NICU-care  *“But you can always stop, right? If you think the treatment is not going to succeed”* |
|  |  |  | It should be established who is allowed to make the decision to stop the AAPT treatment and which criteria will be used for determining when to cease the treatment  *“Who decides when the AAPT is turned off at a certain moment? Because at that point, someone has to make that decision”* |
|  | *Develop a protocol in case the infants passes away during the trial with the AAPT* |  | Consideration should be given to when to discontinue AAPT treatment, ensuring that if the infant's condition deteriorates, they can be with their parents rather than passing away within the AAPT  *“Maybe the infant deteriorates significantly while still in the placenta, whereas the infant could have spent his final moments lying on his mother's chest. You need to be ahead of that moment”* |
|  | *Address logistical considerations how to set up the care around the human AAPT trials* |  | Hospital:   - The operating room - *“Logistics and capacity”* |
|  | *Address logistical considerations regarding centralization of the trials in a limited number of hospitals* | The care with AAPT should be centralized in academic hospitals, so you can control the environment.  *“At that moment, the location doesn't matter to me at all”* | AAPT treatment should be centralized in one center to develop necessary expertise. However, parents may be far from home and there is just one center that will learn how the treatment with the AAPT works.  *“This is high-tech, you know, just like ECMO; we only have it in two centers”* |
|  |  |  | Transport with AAPT should be possible between hospitals.  *“Can you transport an AAPT? […] Because I don't think you would want to transport a pregnant woman with pre-eclampsia by ambulance to another center, right?”* |

***Abbreviations: AAPT = Artificial Amnion and Placenta Technology; NICU = Neonatal Intensive Care Unit.**
